# OCT-based quantitative predictors of coronary dissection during rotational atherectomy in severe calcified lesions

**DOI:** 10.3389/fmed.2025.1640237

**Published:** 2025-08-19

**Authors:** Yu-he Zhou, Hong-li Zhang, Yang Yang, Mei-qin Wang, Jun Pan, Tian Xu, Pei-na Meng, Fei Ye, Yan-qing Wang

**Affiliations:** ^1^Department of General Practice, Jinling Hospital, Affiliated Hospital of Medical School, Nanjing University, Nanjing, China; ^2^The Affiliated Wuxi People’s Hospital of Nanjing Medical University, Wuxi, China; ^3^Division of Cardiology, Nanjing First Hospital, Nanjing Medical University, Nanjing, China

**Keywords:** rotational atherectomy, guidewire bias, coronary dissection, optical coherence tomography, predictive index

## Abstract

**Background:**

In the treatment of coronary calcification by rotational atherectomy (ROTA), guidewire bias is often considered to lead to procedure-associated coronary dissections or perforations. However, the actual meaning of guidewire bias is unclear, though it usually refers to the cross-sectional location of the intravascular imaging (IVI) catheter in the coronary artery. This study tentatively explores the quantitative criteria in optical coherence tomography (OCT) imaging of guidewire bias, which may cause ROTA-induced coronary dissection.

**Methods:**

A total of 21 patients with severe calcified coronary lesions who underwent ROTA treatment were enrolled in our study. OCT successfully detected these patients pre-ROTA and post-ROTA. All observed coronary segments were analyzed cross-sectionally at every 1-mm interval after manual coregistration of pre-ROTA and post-ROTA OCT images. ROTA-related coronary dissection was the primary endpoint.

**Results:**

A total of 388 OCT cross-sectional images were effectively measured and analyzed to assess the distribution and characteristics of plaque and OCT catheter location pre-ROTA, as well as the presence or absence of coronary dissections post-ROTA after manual coregistration. According to the receiver operating characteristic (ROC) analysis, the distance from the center of OCT catheter to the media at the bias direction (D_cmb_) (area under the curve (AUC): 1.000, *p* < 0.001, 95% confidence intervals (CI): 0.999–1.000) and the touch angle (AUC: 0.988, *p* < 0.001, 95%CI: 0.968 to 1.000) were strongly correlated with ROTA-related coronary dissection, with the corresponding cutoff values of 0.720 mm and 98.2° respectively.

**Conclusion:**

D_cmb_ and touch angle detected by OCT are two valuable and convenient independent predictors of ROTA-related coronary intimal dissections caused by guidewire bias.

## Introduction

Guidewire bias is an inevitable occurrence during percutaneous coronary intervention (PCI), especially when the target vessel is tortuous or angulated. However, it is difficult to accurately describe and quantitatively analyze this common phenomenon using only coronary angiography, which can significantly affect the safety and efficacy of PCI procedures, such as rotational atherectomy (ROTA) for severe calcified lesions. With the development of intravascular imaging (IVI) technology in PCI, understanding of guidewire bias has become more in-depth by IVI (including optical coherence tomography (OCT) and intravascular ultrasound (IVUS)) (1). There are, however, no in-depth studies on how guidewire bias affects PCI, especially on calcified lesions. Although rotational atherectomy (ROTA) therapy is one of the more effective treatments for moderate and severe coronary arterial calcified lesions, the current concept of treating coronary calcified lesions by ROTA is modification rather than debulking (2–4). Wire bias may be a negative factor in ROAT treatment (5). A previous small number observational study showed that the effect of ROTA burr on the four vertical directions of the vascular cross-section was different due to the guide wire bias. Based on the IVUS findings, the reduction of intima to media thickness in the vertical direction (IVI contact point) is significantly greater than that in the horizontal direction (6). Deep ROTA procedure can even lead to coronary dissection or perforation (7–9).

Although the concept that guidewire bias can lead to a significant increase in ROTA-related complications of dissection is not new, quantitative indices to support that belief have yet to be published. Even in the current era of popular IVI guiding PCI, the concept of guidewire bias that can easily lead to ROTA-related dissection is still in its infancy. Prior studies have demonstrated that OCT-detected dissections, particularly those extending beyond the intima, are associated with increased risk of peri-procedural myocardial infarction and target vessel revascularization (10). In this study, we focused on intimal dissections as they represent the earliest stage of vascular injury and are most likely to be directly attributable to wire bias rather than procedural manipulation. The purpose of this study was to retrospectively analyze OCT data of patients with moderate to severe coronary calcification pre-ROTA and post-ROTA treatment, and to explore the true meaning of guidewire bias that may easily lead to ROTA-related coronary dissections.

## Methods

### Study design

This was a retrospective, single-center, observational study to explore whether guidewire bias detected by OCT could easily lead to ROTA-related coronary dissections. The quantitative criteria of guidewire bias are further analyzed by comparing OCT data and coronary angiography pre-ROTA and post-ROTA procedure using post manual co-registration correction.

From October 2012 to July 2021, a total of 717 cases of ROTA were screened in the database of Nanjing First Hospital (Nanjing Medical University), among which 21 patients received OCT detection pre-ROTA and post-ROTA procedures. OCT was routinely attempted in all ROTA cases unless contraindicated (e.g., renal insufficiency). The final 21 cases were selected solely based on complete pre-ROTA/post-ROTA OCT data availability, regardless of procedural complexity or outcomes. Exclusions included records that indicated: lesion preparation with predilatation before OCT detection, poor quality of OCT images that could not be measured precisely, coronary angiographic analysis that did not clarify the ROTA burr movement segment, and cases where OCT data were missing pre-ROTA or post-ROTA. Finally, a total of 388 pairs of OCT cross-sectional images were measured and analyzed at 1 mm intervals. This retrospective study was approved by the institutional review board, and written informed consent was obtained from all patients.

The selection of D_cmb_ and touch angle as primary predictors was hypothesis-driven based on: (1) prior IVUS observations showing asymmetric ablation at wire bias sites; (2) pilot data indicating catheter entrapment in subintimal space when these parameters exceeded certain thresholds.

All procedures used ROTA-specific wires (Rotawire™ Floppy, Boston Scientific, 0.009-inch body/0.014-inch tip) with backup guidewires (Fielder XT, Asahi Intecc) when needed for tortuous anatomy.

### Coronary angiography analysis

Quantitative coronary analysis (QCA) was not important in this study because the analysis was based on OCT cross-sectional data rather than changes in coronary artery diameter. The aim of coronary angiography analysis is to clarify the trajectory and range of ROTA burr movement segments and to maintain consistency with OCT analysis.

### OCT image acquisition

OCT images were acquired after nitroglycerin intra-coronary injection into the target coronary artery with moderate or severe calcified lesions pre-ROTA and post-ROTA. OCT image checking with both ILUMIEN OPTIS and C7-XR (LightLab Imaging Incorporated, Westford, MA, USA) was performed using a 2.7F (Dragonfly OPTIS or Dragonfly Duo imaging catheter, Westford, MA, USA) catheter automatic pullback at a speed of 36 mm/s with continuous contrast injection (3–4 mL/s). Due to lesion obstruction, not all calcified lesions’ segments could be detected by OCT before ROTA.

Therefore, in this study, if the OCT catheter passed through the whole ROTA segment before the ROTA operation and the image quality was high enough for analysis, the farthest point of the ROTA burr movement recorded by coronary angiography guidance was the distal mark for analysis. If the OCT catheter could not pass through the whole ROTA segment before the ROTA operation, the endpoint for analysis was marked by the farthest point that the OCT catheter could reach before the ROTA operation. The starting point of analysis was the beginning of the ROTA operation site, as shown by coronary angiography. Pre-ROTA OCT coverage was achieved in 80% of cases (16/21), primarily limited by severe stenosis that prevents catheter passage. These cases were excluded to ensure accurate coregistration analysis.

### OCT images analysis

All OCT data (pre-ROTA and post-ROTA) and coronary angiography were analyzed post manual co-registration based on the range of motion of the ROTA burr according to coronary angiography or range of OCT detection pre-ROTA. Off-line OCT images were analyzed cross-sectionally at every mm interval after manual coregistration of OCT imaging pre-ROTA and post-ROTA by LightLab OPTIS, E. 4 software (LightLab Imaging Incorporated, Westford, MA, USA). Calcification in OCT was defined as a signal-poor and heterogeneous region with sharply delineated near and far boundaries (11–13). Coronary dissections are defined as rims of tissue protruding into the lumen (14, 15), and were classified into five categories (similar to the IVUS classification): intimal: limited to the intima or plaque, and not extending to the media; medial: extending into the media; adventitial: extending through the external elastic membrane (EEM); intramural hematoma: an accumulation of flushing media within the medial space, displacing the internal elastic membrane inward and EEM outward; intra-stent: separation of neointimal hyperplasia from stent struts, usually seen only after treatment of in-stent restenosis (the last category is also outside the scope of this study) (16). Intimal dissection induced by the ROTA procedure only met the requirements of this study.

**Figure 1:**
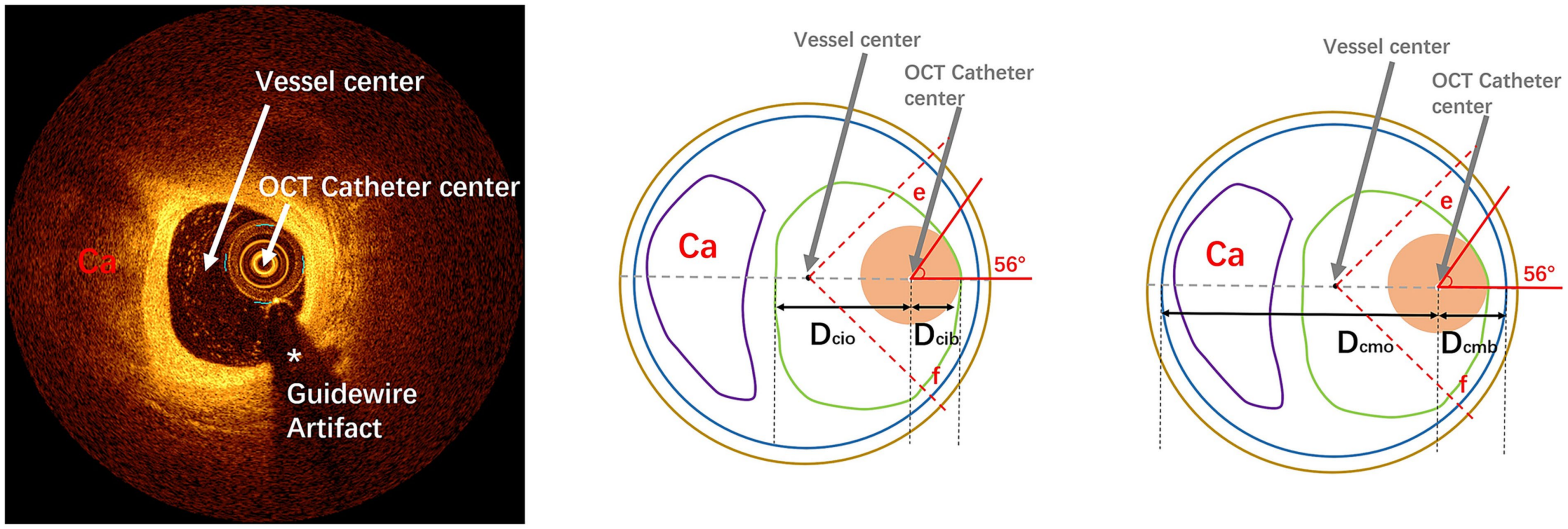
Pattern diagram of cross-sectional parameter measurement by OCT imaging: the left diagram represents an OCT cross-sectional image of calcified lesions, respectively. The upper, middle, and right images represent OCT measurements. In this diagram, the green line represents the intima, the blue line represents the media, the coffee line represents the extravascular, the purple line represents the calcified plaques, and the fleshy circle represents the OCT catheter. D_cib_: distance from center of IVI catheter to intima at the bias direction; D_cio_: distance from center of IVI catheter to intima at the opposite side of the bias; D_cmb_: distance from center of IVI catheter to media at the bias direction; D_cmo_: distance from center of IVI catheter to media at the opposite side of the bias. The fan-shaped area between line e and line f (45° from the line between the center of the IVI catheter and the center of the vessel) is defined as the guidewire bias quadrant. OCT, optical coherence tomography; IVI, intravascular imaging.

**Figure 2:**
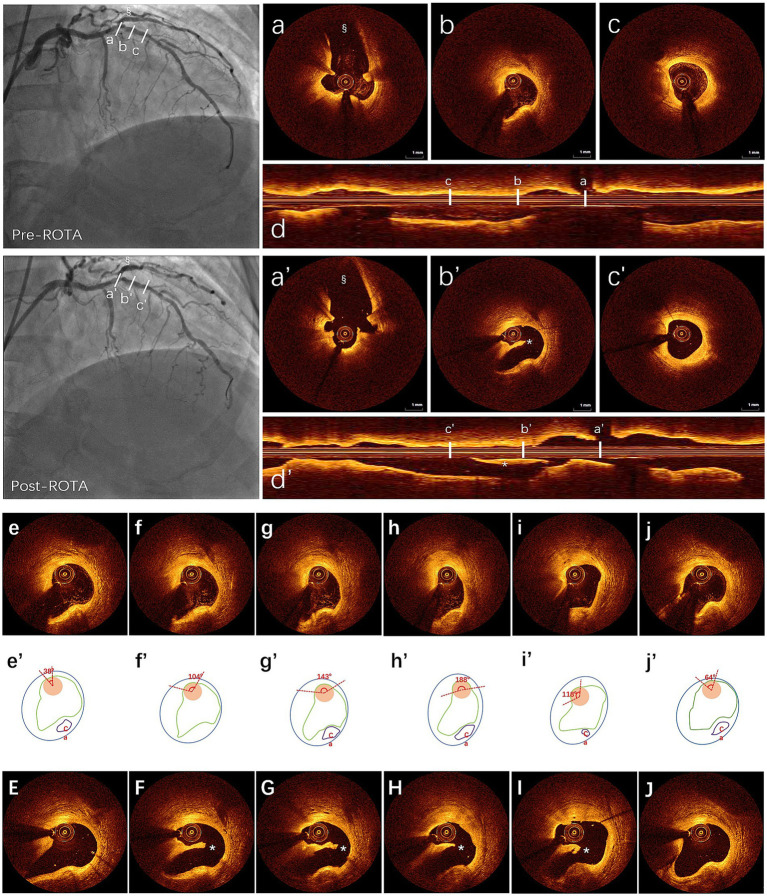
ROTA-related dissection measured by OCT post manual coregistration: first row: **(a–c)** three cross-sectional OCT images detection to identify wire bias pre-ROTA shown by angiography, in the top left, **(a)** side branch ostium for manual coregistration; **(b)** guidewire bias (OCT catheter bias to normal vessel wall); **(c)** non-guidewire bias; **(d)** longitudinal view of OCT pre-ROTA. Second row: **(a’–c’)** same layers of cross-sectional OCT images post-ROTA after manual coregistration with same side branch in which angiography showed a haziness result in the below left, **(a’)** side branch; **(b’)** intimal dissection with OCT catheter trapped beneath the intima (subintimal space); **(c’)** without dissection; **(d’)** longitudinal view of OCT post-ROTA. *(b’,d’) showed ROTA-related coronary dissection. ^§^(angiography pre- and post-ROTA, **a,a’**) showed side branch. OCT catheter bias to nearly normal vessel pre-ROTA **(b)** and intimal dissection occurred post-ROTA with OCT catheter trapped beneath the dissected intimal **(b’)** in the guidewire bias quadrant. The third to fifth row: The third row shows the different catheter contact angles with vessel wall based on continuous OCT detection **(e–j)**, the fourth row of the schematic diagram shows the measurement method of the contact angle **(e’–j’)**, and the third row shows the location of the procedure related coronary dissection after manual coregistration of OCT findings pre-ROTA and post-ROTA **(E–J)**. OCT, optical coherence tomography; ROTA, rotational atherectomy.

Only intimal dissections were included as primary endpoints because: (1) medial/adventitial dissections often result from complex interactions between plaque morphology and device manipulation rather than wire bias alone; (2) intimal disruption is the most common ROTA-related complication reported in IVUS/OCT studies (17). Pre-ROTA/post-ROTA OCT images and coronary angiography were manually coregistered by fiduciary side branch based on the ROTA burr route before measurement. Each measurement index of OCT at each cross-section of the corresponding segment in this study was defined as follows: at each cross-section, the center point of the OCT catheter is connected to the center point of the vessel in a straight line. The quadrant of the OCT catheter bias in the fan-shaped face with the vascular center as the dot is defined as the bias quadrant (Figure 1), as per the center line, and then each index was measured. (1) Distance from the center of OCT catheter to intima at the bias direction (D_cib_); (2) distance from the center of OCT catheter to intima at the opposite side of the bias (D_cio_); (3) distance from the center of OCT catheter to media at the bias direction (D_cmb_); (4) distance from the center of OCT catheter to media at the opposite side of the bias (D_cmo_) (Figure 1); (5) touch angle: the arc of contact between OCT catheter and the intima of coronary artery (Figures 1, 2); (6) luminal area (LA): the area bounded by the luminal border; (7) minimal luminal diameter (M_in_LD): the shortest length through the center point of the lumen; (8) maximal luminal diameter (M_ax_LD): the longest length through the center point of the lumen; (9) lumen eccentricity (LE): defined as (M_ax_LD-M_in_LD)/ M_ax_LD; (10) EEM cross sectional area (CSA); (11) average vessel diameter (AVD): defined as (minimal EEM diameter + maximal EEM diameter)/2. All the image data were analyzed offline for ROTA burr movement segment by two independent professional analysts who were blinded to the other analyses as well as to the ROTA procedure and corresponding coronary angiographic result. Finally, OCT data were compared cross-section by cross-section to confirm the relationship of wire bias to ROTA-related coronary dissection by OCT detection. Analysts were fully blinded to patient identifiers, angiographic findings, and procedural outcomes during OCT measurements. Disagreements were adjudicated by a third independent reviewer using pre-specified criteria.

### Statistical analysis

Before statistical analysis of the data, intra-observer and inter-observer variability in the image analysis were assessed by evaluating 50 randomly selected cross-sectional OCT slides. Consistency test was performed by Kappa statistics for categorical variables or intraclass correlation coefficients (ICC) for continuous variables. Categorical variables were expressed as frequencies and were compared using chi-squared statistics or Fisher’s exact test as appropriate. Continuous variables were expressed as the mean ± standard deviation for the normal distribution using the Kolmogorov–Smirnov test or shown as median and first and third quartiles and compared using the Mann–Whitney U-test or Kruskal–Wallis test with *post hoc* analysis using Dunn–Bonferroni test for non-normally distributed continuous variables. After univariate correlation analysis, for the identification of potential correlations between variables, receiver operating characteristic (ROC) analysis was used to determine the discriminatory capability as an area under the curve (AUC) with the optimal cutoff value using Youden’s index [the maximum value of (sensitivity + specificity – 1)]. With 56 events and 2 primary predictors, our analysis meets the conventional 10–20 events per variable rule for logistic regression. Bootstrap internal validation showed <5% optimism in AUC estimates. All tests were two-tailed with a 0.05 significance level. All statistical analyses, except Passing-Bablok regression, were performed with SPSS 18.0 (IBM, Armonk, New York) and Windows version R 4.0.5 software.^1^ As a sensitivity analysis, we performed generalized estimating equation (GEE) models accounting for within-patient clustering, which yielded consistent results with the primary ROC analysis.

## Results

Consistency test between inter-observer and intra-observer variability for OCT images measurement.

There was very good inter-observer and intra-observer agreement for the OCT measurement of D_cib_ (ICC: 0.996, 0.998), D_cio_ (ICC: 0.990, 0.999), D_cmb_ (ICC: 0.992, 0.992), and D_cmo_ (ICC: 0.957, 0.997); and agreement for the assessment of coronary intimal dissection by OCT detection (Kappa: 0.878, 0.878), plaque characteristics (with or without calcification) at wire bias direction (Kappa: 0.961,0.985).

### Patients’ clinical characteristics and basic coronary lesions’ location

The baseline clinical and coronary disease characteristics were summarized in Table 1. The average patient age was 71 ± 7.20, and 76.2% were men. The prevalence of chronic kidney disease was only 4.7%, and the most common target vessel for ROTA treatment was the left anterior descending artery (LAD), approximately 81.0% and located at the proximal segment, approximately 42.9% (Table 1).

**Table 1:** Baseline clinical and coronary disease characteristics of patients.

Variables	*N* = 21
Age, years	71 ± 7.20
Male, *n* (%)	16 (76.2)
Body mass index, kg/m^2^	24.11 ± 3.97
Diabetes mellitus, *n* (%)	4 (19.0)
Hypertension, *n* (%)	14 (66.7)
Hyperlipidemia, *n* (%)	12 (57.1)
Current smoking, *n* (%)	8 (38.1)
Chronic kidney disease, *n* (%)	1 (4.7)
Previous myocardial infarction, *n* (%)	3 (14.3)
Clinical presentation	
STEMI, *n* (%)	2 (9.5)
Non-STEMI, *n* (%)	0 (0.0)
Unstable angina, *n* (%)	13 (61.9)
Left ventricular ejection fraction, %	62.10 ± 5.32
Total cholesterol, mmol/L	3.54 ± 0.69
Low-density lipoprotein, mmol/L	1.86 ± 0.49
High-density lipoprotein, mmol/L	1.07 ± 0.37
eGFR, mL/min/1.73 m^2^	86.23 ± 25.09
Target vessel	
LAD (%)	17 (81.0)
RCA (%)	3 (14.3)
LCx (%)	1 (4.7)
Lesion location	
Proximal	9 (42.9)
Middle	11 (52.4)
Distal	1 (4.7)
Reference vessel diameter	3.18 ± 0.47
Final burr size	1.65 ± 0.20
BTV	0.53 ± 0.08

Baseline characteristics of 21 ROTA-treated patients. Data shown as mean ± SD or % (*n*/*N*). BTV, Burr-to-vessel ratio (final burr diameter/reference vessel diameter); eGFR, Estimated glomerular filtration rate (mL/min/1.73m^2^); LAD/LCx/RCA, Left anterior descending/left circumflex/right coronary arteries; STEMI, ST-elevation myocardial infarction. Proximal/middle/distal lesion locations are defined by the American College of Cardiology/American Heart Association segment classification. Vessel angulation was quantitatively analyzed in all cases (mean 32° ± 14° by quantitative coronary angiography), with no significant difference between dissection/non-dissection groups (*p* = 0.18). ROTA parameters followed institutional protocol (burr speed 140–160 krpm, gradual burr-upsizing).

Values are median (interquartile range) or % (number of observations/total number of patients). BTV, burr to vessel; eGFR, estimated glomerular filtration rate; EI, expansion index; IVUS, intravascular ultrasound; LAD, left anterior descending artery; LCx, left circumflex; RCA, right coronary artery; STEMI, ST-segment elevation myocardial infarction.

### Measurement of cross-sectional parameters of the OCT catheter bias

A total of 21 segments in 21 patients were included in the OCT image analysis. Ultimately, 388 pairs of cross-sectional OCT slides were compared after manual coregistration of pre-ROTA and post-ROTA images, guided by the angiographic ROTA range. Finally, ROTA-related coronary intimal dissections were found in 56 layers post-ROTA from OCT data. Given that most univariate correlation analyses for potential parameters may indicate wire bias degree. D_cib_, D_cio_, D_cmb_, D_cmo_, final burr size, touch angle, M_in_LD, and M_ax_LD may correlate with ROTA-related coronary intimal dissection. ROC analysis was performed to determine potential relationships involved and further test the discriminatory power of this procedure. The AUC, sensitivity, and specificity were calculated by the R package “pROC.” DeLong’s test was also performed for the optimum predictive index of ROTA-related dissection with two correlated comparative ROC curves. The AUC of D_cmb_ and touch angle was significantly higher than that of other parameters (*p* < 0.001). In terms of clinical convenience, D_cmb_ and touch angle were the best parameters that could predict ROTA-related coronary intimal dissection (Table 2, Figure 2).

**Table 2:** Univariate and ROC analyses for correlation between the variables and ROTA-related coronary intimal dissection.

Variables	Univariate analysis		ROC analysis							
	*r*	*p*	AUC	95%CI		Sensitivity	Specificity	Cutoff value	*p*	YI
D_cib_	−0.407	<0.001	0.942	0.920	0.964	86.4%	95.4%	0.435	<0.001	0.828
D_cio_	0.139	0.006	0.664	0.592	0.737	67.9%	65.1%	1.125	<0.001	0.330
D_cmb_	−0.625	<0.001	1.000	0.999	1.000	98.8%	100.0%	0.720	<0.001	0.988
D_cmo_	0.169	0.001	0.661	0.584	0.738	50.0%	80.7%	2.875	<0.001	0.307
Final burr size	−0.133	0.009	0.581	0.510	0.652	–	–	–	0.053	–
Touch angle	0.912	<0.001	0.988	0.968	1.000	94.6%	98.2%	98.2	<0.001	0.946
M_in_LD	−0.104	0.040	0.540	0.459	0.621	–	–	–	0.343	–
M_ax_LD	−0.119	0.019	0.566	0.495	0.637	–	–	–	0.112	–

Univariate and ROC analyses for dissection predictors. AUC: Area under ROC curve (95%CI). D_cib_/D_cio_/D_cmb_/D_cmo_: Distances defined in Figure 1. M_ax_LD/M_in_LD: Lumen diameters (mm). YI: Youden’s index (sensitivity + specificity − 1). Optimal cutoffs: D_cmb_ = 0.720 mm (sensitivity 98.8%, specificity 100%), touch angle = 98.2° (94.6%/98.2%). *p*-values from DeLong’s test for AUC comparisons.

The coincidence rate of ROTA-related coronary intimal dissection radial distribution involving OCT catheter bias quadrant pre-ROTA was 100% by comparing OCT data pre-ROTA and post-ROTA. Further analysis showed that the bias of the OCT catheter to the coronary dissection (subintimal) was 100% post-ROTA (Figure 2).

Among 21 patients, 30-day clinical follow-up was available for all cases (no deaths/MI/target vessel revascularization). OCT follow-up at 6 months was performed in 8 patients (38.1%), showing complete healing of intimal dissections without late lumen loss.

## Discussion

The main findings in our retrospective study were as follow: (1) As an OCT indicator for the reaction of guidewire bias, D_cmb_ and touch angle are two very valuable and convenient independent predictors of ROTA-related coronary intimal dissections; (2) The OCT catheter bias quadrant pre-ROTA was highly consistent with the quadrant where the ROTA-related coronary intimal dissection was present; (3) OCT catheters are always trapped beneath the ROTA-related dissection. With tortuosity and calcification, the wire will cheese cut through the intima and wall into the subintimal space in a straight fashion, and any equipment introduced will follow this wire.

The operation of ROTA is accomplished by pushing a burr in and out of the calcified lesion along the special guide wire (18, 19). Due to the principle of differential cutting and friction, it is generally believed that ROTA only ablates inelastic fibrocalcific plaques while sparing adjacent elastic tissue that deflects away from the ablating burr (20, 21). However, the ROTA burrs may have ablative effect on the substance in contact (8, 22, 23). Guidewire bias position in an angled or tortuous coronary artery is inevitable: a divergence from the central axis of the vessel or the lumen and may result in ablation on the bias side of the coronary wall occurs, iatrogenic injury to intima or media may result and deeper cutting action could lead to more complications, such as dissection or even perforation (5, 6, 23).

In our retrospective study, we analyzed for the first time the quantitative indicators that guide wire bias caused the motion path bias of ROTA burrs and further led to the occurrence of iatrogenic coronary dissection. Guidewire position bias in an angled or tortuous coronary artery is inevitable; a divergence from the central axis of the vessel or the lumen may result in ablation on the biased side of the coronary wall. If the bias to relatively normal coronary wall occurs, iatrogenic injury to intima or media may result, and deeper cutting action could lead to more complications, such as dissection or even perforation (5, 6, 23). To our knowledge, no quantitative data on this topic have been reported previously. In this study, we used a novel method to analyze the distribution characteristics of coronary cross-sectional plaques, OCT catheter location, and their relationship with coronary dissection post-ROTA using the IVI technique at 1-mm intervals. After manual co-registration of pre-ROTA, post-ROTA, and angiographic data. While plaque morphology and rotational speed may influence dissection risk, our standardized institutional protocols minimized variability in these parameters. Although D_cmb_ and touch angle could predict ROTA-related dissection to the same extent, the simpler and more clinically feasible indicators are D_cmb_ and touch angle. The cutoff value for D_cmb_ was 0.720 mm, which includes the radius of the OCT catheter (0.45 mm) and the thickness of the intima and part of the media. The cutoff value for touch angle was 98.2°.

Shorter D_cmb_ was associated with a higher incidence of ROTA-related coronary intimal dissection, often indicating that the OCT catheter was biased toward and in contact with the coronary vessel wall.

Touch angle is another novel finding that indicates the closeness of the OCT catheter contact with a coronary wall. The bigger the touch angle, the closer to contact; the presence of OCT catheter entrapment in the coronary wall (like a finger pressure sign) is often indicative of relatively soft vessel wall at the bias site (usually normal blood vessel segments rather than calcified lesions), as well as a strong compression force of OCT catheter against the vessel wall.

Sometimes media is not easily recognized at the bias site without a large plaque burden. Touch angle, which can be measured easily, is a good alternative index for avoiding ROTA-related complications (Figure 2).

The following four points must be understood when we use the guidewire bias idea to infer the point of view of ROTA bias:

1. Guidewire bias is the starting point, but cannot be quantified and guided to optimize ROTA precisely.
2. Catheter bias can be quantified by IVI checking along a PCI guidewire of 0.014 inch, but IVI is a monorail system (not an over-the-wire system like ROTA), so it may be similar to or consistent with the guidewire position.
3. A special ROTA wire (0.009-inch body and 0.014-inch tip) was replaced during ROTA operation, which might affect guidewire bias by different support and compliance.
4. Different positions between the tip of the PCI guidewire and the ROTA wire, and the placement of the guide catheter may be changed, which may result in the inaccurate alignment between the ROTA burr movement route and the IVI catheter.

However, the condition of guidewire bias due to coronary anatomical characteristics (distortion and angulation, etc.) will not change. In most cases, IVI bias can reflect the uneven effect of ROTA, especially when the bias is in normal vascular segments, which easily leads to ROTA-related coronary dissections.

Our study showed that the quadrant of pre-ROTA OCT catheter bias was highly consistent (100%) with the location of ROTA-related dissections, indicating a strong association between the position of the ROTA burr and the OCT catheter placement.

However, wire bias could not be detected by angiography precisely unless IVI detection was performed; wire position appears to have been disturbed by the IVI catheter, and the concept of wire bias may, upon further study, be replaced by IVI catheter bias.

Another critical finding was that the OCT catheter was always trapped beneath the intima (subintimal space) if ROTA-related dissection had occurred. This has potential clinical implications, as further replacement of a larger ROTA burr size may lead to further expansion and deepening of the dissection, and even perforation. So, the most important idea is to recognize ROTA-related dissection as early as possible and avoid further ROTA (24). Coronary dissection caused by ROTA must be treated seriously and should be discontinued ROTA procedure once detected, otherwise more serious complications such as inhibited flow or coronary perforation may occur (25). The sensitivity of angiographic ROTA-related coronary dissection was significantly lower than that of IVI detection, and even if the dissections were detected by angiography, it was not possible to determine whether the guidewire bias was beneath the dissections (subintimal space), so IVI-guided ROTA therapy was recommended to detect the ROTA-related dissection early and accurately. While manual coregistration showed excellent inter-observer agreement in our core lab, its real-world reproducibility requires clear fiduciary markers (e.g., side branches) and standardized measurement protocols. Future AI-based auto-coregistration tools could mitigate operator dependency.

The 0.72 mm Dcmb cutoff corresponds to: (1) OCT catheter radius (0.45 mm) plus normal intimal thickness (0.2–0.3 mm) and (2) the point where >50% of the catheter surface contacts the wall. In practice, exceeding this threshold should prompt: (a) wire repositioning, (b) reduced burr-to-artery ratio, or (c) switch to orbital atherectomy in high-risk cases.

## Limitations

First, although this study represents one of the largest OCT-guided ROTA cohorts to date, the sample size (*n* = 21) remains modest, and all data were collected from a single high-volume center. Multi-center validation is needed to confirm generalizability. Second, coronary dissection derived by ROTA is caused not only by wire bias but also by manipulation. Third, OCT detection for the whole lesion segment was less than 80% (16 cases) pre-ROTA in our study, which means nearly one in four patients. Even with OCT checking before ROTA, it is still not known whether guidewire bias is due to the heavy stenosis that the OCT catheter cannot pass through. The 20% exclusion rate due to incomplete OCT coverage may bias results toward less complex lesions, though this reflects real-world technical constraints. Moreover, the requirement for complete OCT imaging may have introduced selection bias toward less tortuous vessels where the OCT catheter could traverse the entire lesion. Fourth, in this study, BTV did not draw the conclusion that it was related to the ROTA-related dissection correlation, which may be due to the low overall BTV data in this study. Fifth, although primary analyses did not adjust for within-patient clustering, sensitivity analyses with GEE confirmed result robustness. Sixth, we could not adjust for rotational speed or burr size due to uniform institutional protocols, though these may influence dissection risk. Seventh, limited long-term OCT follow-up data preclude assessment of delayed healing patterns.

In our forthcoming research, we intend to undertake a prospective study, aiming to predict postoperative complications by assessing Dcmb and touch angle parameters. Once we further corroborate the perspectives put forth in this paper, we will delve into the potential implications.

## Conclusion

D_cmb_ and touch angle detected by OCT are valuable and convenient independent predictors of ROTA-related coronary intimal dissections caused by guidewire bias.

## Data Availability

The datasets presented in this study can be found in online repositories. The names of the repository/repositories and accession number(s) can be found in the article/Supplementary material.

## Footnotes

^1^ https://www.r-project.org/

## Author’s note

The authors acknowledge that an earlier version of this manuscript was inadvertently posted as a preprint (https://europepmc.org/article/ppr/ppr414193). The preprint version contained preliminary analyses without coregistration validation or ROC cutoff derivation, and it has not undergone peer review. The current manuscript includes substantially revised analyses and has only been formally submitted to this journal for evaluation.

## Supplementary material

SUPPLEMENTARY FIGURE 1 OCT-guided rotational atherectomy workflow: procedural decision algorithm for preventing ROTA-related dissections. OCT, optical coherence tomography; ROTA, rotational atherectomy.

## References

[1] Danson EJ Hansen P Bhindi R. Wire bias in coronary measurement using optical coherence tomography. Cardiovasc Interv Ther. (2018) 33:217–23. doi: 10.1007/s12928-017-0468-7, PMID: 28540635

[2] Uetani T Amano T. Current status of rotational atherectomy in the drug-eluting stent era. Circ J. (2018) 82:946–7. doi: 10.1253/circj.CJ-18-0170, PMID: 29515046

[3] Tomey MI Kini AS Sharma SK. Current status of rotational atherectomy. JACC Cardiovasc Interv. (2014) 7:345–53. doi: 10.1016/j.jcin.2013.12.196, PMID: 24630879

[4] You W Wu XQ Ye F Chen SL. Advantages of transradial rotational atherectomy versus transfemoral approach in elderly patients with hard-handling calcified coronary lesions–a single center experience. Acta Cardiol Sin. (2018) 34:464–71. doi: 10.6515/ACS.201811_34(6).20180427A, PMID: 30449986 PMC6236566

[5] Sakakura K Ito Y Shibata Y Okamura A Kashima Y Nakamura S. Correction to: clinical expert consensus document on rotational atherectomy from the Japanese association of cardiovascular intervention and therapeutics. Cardiovasc Interv Ther. (2021) 36:19–9. doi: 10.1007/s12928-020-00749-0, PMID: 33389647 PMC7829231

[6] Oishi Y Okamoto M Sueda T Hashimoto M Karakawa S Kambe M. Guidewire bias in rotational atherectomy in the angled lesion evaluation based on the thickness of the ablated intima and media. Circ J. (2002) 66:659–64. doi: 10.1253/circj.66.659, PMID: 12135134

[7] Wang YH Chen WJ Chen YW Lai CH Su CS Chang WC. Incidence and mechanisms of coronary perforations during rotational atherectomy in modern practice. J Interv Cardiol. (2020) 2020:1–9. doi: 10.1155/2020/1894389, PMID: 33223973 PMC7673942

[8] Hashimoto K Fujii K Shibutani H Matsumura K Tsujimoto S Otagaki M. Prediction of optimal debulking segments before rotational atherectomy based on pre-procedural intravascular ultrasound findings. Int J Cardiovasc Imaging. (2021) 37:803–12. doi: 10.1007/s10554-020-02080-4, PMID: 33111175

[9] Kini AS Vengrenyuk Y Pena J Motoyama S Feig JE Meelu OA. Optical coherence tomography assessment of the mechanistic effects of rotational and orbital atherectomy in severely calcified coronary lesions. Catheter Cardio Interv. (2015) 86:1024–32. doi: 10.1002/ccd.26000, PMID: 25964009

[10] Ozaki Y Taniguchi M Katayama Y Ino Y Tanaka A. Early healing of spontaneous coronary artery dissection confirmed by optical coherence tomography. J Invasive Cardiol. (2023) 35:E154–5. PMID: 36884362 10.25270/jic/22.00195

[11] Räber L Mintz GS Koskinas KC Johnson TW Holm NR Onuma Y. Clinical use of intracoronary imaging. Part 1: guidance and optimization of coronary interventions. An expert consensus document of the European Association of Percutaneous Cardiovascular Interventions. Eur Heart J. (2018) 39:3281–300. doi: 10.1093/eurheartj/ehy285, PMID: 29790954

[12] Mintz GS. Intravascular imaging of coronary calcification and its clinical implications. JACC-Cardiovasc Imaging. (2015) 8:461–71. doi: 10.1016/j.jcmg.2015.02.003, PMID: 25882575

[13] Tearney GJ Regar E Akasaka T Adriaenssens T Barlis P Bezerra HG. Consensus standards for acquisition, measurement, and reporting of intravascular optical coherence tomography studies: a report from the International Working Group for Intravascular Optical Coherence Tomography Standardization and Validation. J Am Coll Cardiol. (2012) 59:1058–72. doi: 10.1016/j.jacc.2011.09.079, PMID: 22421299

[14] Prati F Cera M Ramazzotti V Imola F Giudice R Albertucci M. Safety and feasibility of a new non-occlusive technique for facilitated intracoronary optical coherence tomography (OCT) acquisition in various clinical and anatomical scenarios. EuroIntervention. (2007) 3:365–70. doi: 10.4244/EIJV3I3A66, PMID: 19737719

[15] Prati F Regar E Mintz GS Arbustini E Di Mario C Jang IK. Expert review document on methodology, terminology, and clinical applications of optical coherence tomography: physical principles, methodology of image acquisition, and clinical application for assessment of coronary arteries and atherosclerosis. Eur Heart J. (2010) 31:401–15. doi: 10.1093/eurheartj/ehp433, PMID: 19892716

[16] Fujii K Kubo T Otake H Nakazawa G Sonoda S Hibi K. Expert consensus statement for quantitative measurement and morphological assessment of optical coherence tomography. Cardiovasc Interv Ther. (2020) 35:13–8. doi: 10.1007/s12928-019-00626-5, PMID: 31602597

[17] Deng C Liu Z Zhang W Deng Y Liu H Bai Z. Comparison of Neoatherosclerosis and neovascularization of restenosis after drug-eluting stent implantation: an optical coherence tomography study. Rev Cardiovasc Med. (2023) 24:341. doi: 10.31083/j.rcm2412341, PMID: 39077084 PMC11272882

[18] Fourrier JL Bertrand ME Auth DC Lablanche JM Gommeaux A Brunetaud JM. Percutaneous coronary rotational angioplasty in humans: preliminary report. J Am Coll Cardiol. (1989) 14:1278–82. doi: 10.1016/0735-1097(89)90428-2, PMID: 2808983

[19] Reifart N Vandormael M Krajcar M Göhring S Preusler W Schwarz F. Randomized comparison of angioplasty of complex coronary lesions at a single center: excimer laser, rotational Atherectomy, and balloon angioplasty comparison (ERBAC) study. Circulation. (1997) 96:91–8. doi: 10.1161/01.CIR.96.1.91, PMID: 9236422

[20] Barbato E Shlofmitz E Milkas A Shlofmitz R Azzalini L Colombo A. State of the art: evolving concepts in the treatment of heavily calcified and undilatable coronary stenoses-from debulking to plaque modification, a 40-year-long journey. EuroIntervention. (2017) 13:696–705. doi: 10.4244/EIJ-D-17-00473, PMID: 28844031

[21] O'Neill WW. Mechanical rotational atherectomy. Am J Cardiol. (1992) 69:F12–8. doi: 10.1016/0002-9149(92)91177-6, PMID: 1621645

[22] Kim SS Yamamoto MH Maehara A Sidik N Koyama K Berry C. Intravascular ultrasound assessment of the effects of rotational atherectomy in calcified coronary artery lesions. Int J Cardiovasc Imaging. (2018) 34:1365–71. doi: 10.1007/s10554-018-1352-y, PMID: 29663177

[23] Iannopollo G Gallo F Mangieri A Laricchia A Erriquez A Tzanis G. Tips and tricks for rotational atherectomy. J Invasive Cardiol. (2019) 31:E376–83. PMID: 31786529 10.25270/jic/19.3112.E376

[24] de Belder AJ. Rotational atherectomy: re-emergence of an old technique. Heart. (2018) 104:440–8. doi: 10.1136/heartjnl-2016-310319, PMID: 28500242

[25] Barbato E Carrié D Dardas P Fajadet J Gaul G Haude M. European expert consensus on rotational atherectomy. EuroIntervention. (2015) 11:30–6. doi: 10.4244/EIJV11I1A6, PMID: 25982648

