# Supplementary figures and images for: OCT-based quantitative predictors of coronary dissection during rotational atherectomy in severe calcified lesions
Source: Front Med (Lausanne). 2025 Aug 19;12:1640237. doi: 10.3389/fmed.2025.1640237 (PMC12401907; doi:10.3389/fmed.2025.1640237)

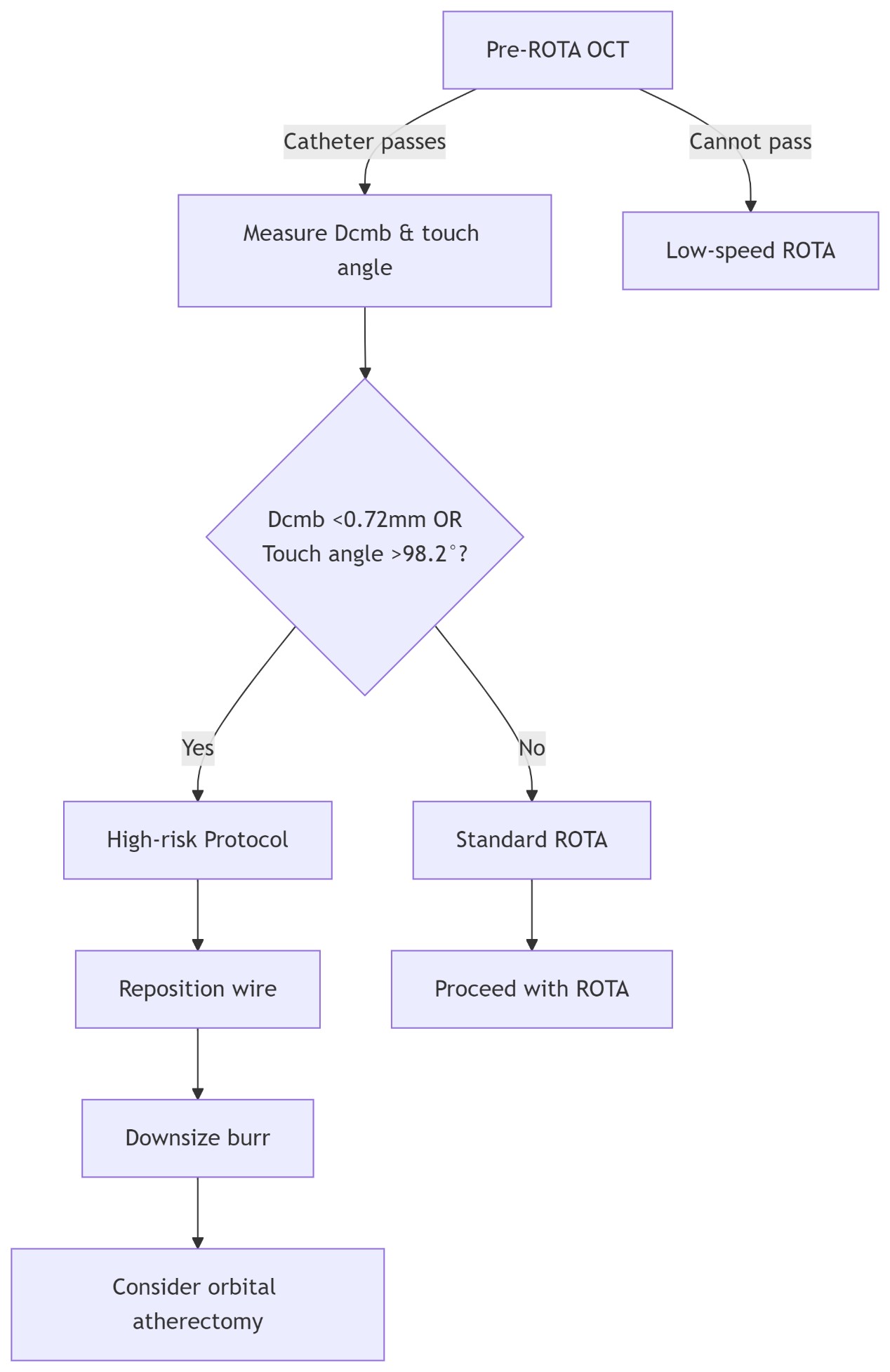

Supplement: SUPPLEMENTARY FIGURE 1 — OCT-guided rotational atherectomy workflow: procedural decision algorithm for preventing ROTA-related dissections. OCT, optical coherence tomography; ROTA, rotational atherectomy. [file Image_1.jpg]
